# Use of Electronic Medical Records to Estimate Changes in Pregnancy and Birth Rates During the COVID-19 Pandemic

**DOI:** 10.1001/jamanetworkopen.2021.11621

**Published:** 2021-06-03

**Authors:** Molly J. Stout, Cosmas J. M. Van De Ven, Vikas I. Parekh, Jennifer L. Pardo, Maxim Garifullin, Min Xu, Dee E. Fenner, Roger D. Smith

**Affiliations:** 1 Department of Obstetrics and Gynecology, University of Michigan, Ann Arbor; 2 Department of Internal Medicine, University of Michigan, Ann Arbor; 3 Office of Capacity Operations, University of Michigan, Ann Arbor

## Abstract

**Importance:**

The influence of the COVID-19 pandemic on fertility rates has been suggested in the lay press and anticipated based on documented decreases in fertility and pregnancy rates during previous major societal and economic shifts. Anticipatory planning for birth rates is important for health care systems and government agencies to accurately estimate size of economy and model working and/or aging populations.

**Objective:**

To use projection modeling based on electronic health care records in a large US university medical center to estimate changes in pregnancy and birth rates prior to and after the COVID-19 pandemic societal lockdowns.

**Design, Setting, and Participants:**

This cohort study included all pregnancy episodes within a single US academic health care system retrospectively from 2017 and modeled prospectively to 2021. Data were analyzed September 2021.

**Exposures:**

Pre– and post–COVID-19 pandemic societal shutdown measures.

**Main Outcomes and Measures:**

The primary outcome was number of new pregnancy episodes initiated within the health care system and use of those episodes to project birth volumes. Interrupted time series analysis was used to assess the degree to which COVID-19 societal changes may have factored into pregnancy episode volume. Potential reasons for the changes in volumes were compared with historical pregnancy volumes, including delays in starting prenatal care, interruptions in reproductive endocrinology and infertility services, and preterm birth rates.

**Results:**

This cohort study documented a steadily increasing number of pregnancy episodes over the study period, from 4100 pregnancies in 2017 to 4620 in 2020 (28 284 total pregnancies; median maternal [interquartile range] age, 30 [27-34] years; 18 728 [66.2%] White women, 3794 [13.4%] Black women; 2177 [7.7%] Asian women). A 14% reduction in pregnancy episode initiation was observed after the societal shutdown of the COVID-19 pandemic (risk ratio, 0.86; 95% CI, 0.79-0.92; *P* < .001). This decrease appeared to be due to a decrease in conceptions that followed the March 15 mandated COVID-19 pandemic societal shutdown. Prospective modeling of pregnancies currently suggests that a birth volume surge can be anticipated in summer 2021.

**Conclusions and Relevance:**

This cohort study using electronic medical record surveillance found an initial decline in births associated with the COVID-19 pandemic societal changes and an anticipated increase in birth volume. Future studies can further explore how pregnancy episode volume changes can be monitored and birth rates projected in real-time during major societal events.

## Key Points

**Question:**

Can electronic health care records be used to monitor and project changes in pregnancy and birth rates after the COVID-19 pandemic societal shutdown?

**Findings:**

In this cohort study of pregnancies within a large US university health care system, a model using electronic medical records (used retrospectively from 2017 and modeled prospectively to 2021) projected an initial decline in births associated with the COVID-19 pandemic societal shutdown, predominantly related to fewer conceptions following the societal changes instituted to control COVID-19 spread. This decline was followed by a projected birth volume surge anticipated to occur in summer 2021.

**Meaning:**

These findings suggest that electronic medical records can be used to model and project birth volume changes and demonstrate that the COVID-19 pandemic societal changes are associated with reproductive choices.

## Teaser

This cohort study of all pregnancies in a large US university health care system examines changes in birth volumes associated with the COVID-19 societal shutdown and models prospective trends for birth volume post–societal shutdown.

## Introduction

The COVID-19 pandemic and associated societal measures to control the spread of the virus have brought about significant changes in almost every aspect of life in the US and globally. The economic and societal effects of the pandemic are vast, with consequences not only for large systems such as health care and education, but also for individuals and families. Previous large societal disruptions in the US, such as the 1918 H1N1 Influenza pandemic,^1^ Great Depression (1929),^2^ and Great Recession (2008)^3^ have influenced population growth and fertility rates, but the exact effects of COVID-19 on fertility and birth rates are speculative to date. In the US, a multitude of factors could variably influence pregnancy rates. For example, economic concerns may lead people to postpone conception, whereas decreased access to contraceptive services could lead to increased unintended pregnancy rates. A 2020 Guttmacher Institute study^4^ demonstrated that 40% of women reported changes in plans for childbearing due to the COVID-19 pandemic, 41% of women with children reported worry about being able to take care of their children, and 33% of women had to delay or cancel an appointment for reproductive health or contraception care. Additionally, the COVID-19 pandemic has highlighted longstanding economic and health disparities in the US,^4,5,6^ yet how such disparities will influence fertility rates and obstetric complications are unknown at this time.

Anticipatory planning for birth rates is important for health care systems to appropriately anticipate increasing or decreasing staffing needs and patient volumes. Population size and population dynamics are of interest to economists to document size of economy and model working and/or aging populations. Often, the consequences of major societal events such as economic and natural disasters or infection pandemics are documented only after the fact or as decreasing birth rates are noted. At the University of Michigan Hospital, we use projection modeling and active management of estimated date of deliveries (EDD) to control obstetric birth volumes in our system and anticipate staffing needs for our birth center. In this analysis, we applied our EDD management modeling techniques to project anticipated delivery volumes after the COVID-19 pandemic and describe the projected decrease in birth rates from our center.

## Methods

This observational cohort study including all pregnancy episodes within the health care system of the University of Michigan Hospital, a large US academic hospital, examined birth rates retrospectively from 2017 through current pregnancy episodes and modeled them prospectively to October 2021. The primary exposure was the COVID-19 pandemic societal shutdown. The stay-at-home order in Michigan was placed on March 15, 2020. Our primary outcome of interest was the start of pregnancy episodes within our health care system, trends in the volume of pregnancies, and evaluation of potential explanations for these volume changes. This analysis was approved by the University of Michigan institutional review board, which exempted the study because it used deidentified data kept by the system for institutional quality improvement, and deemed that no direct informed consent from individual patients was required. Data reporting and analyses followed the Strengthening the Reporting of Observational Studies in Epidemiology (STROBE) reporting guideline for cohort studies.

Pregnancy episodes begin with a patient-initiated contact with our health care system to request pregnancy-related care, such as initial prenatal visit or ultrasound for pregnancy dating. Pregnancy episodes remain open until 1 of the following events occurs: (1) delivery, (2) documented pregnancy loss (ie, miscarriage, ectopic, etc), or (3) no contact with the patient for 90 days after the EDD. The final EDD was determined by best obstetric estimate^7^ based on last menstrual period (LMP) and ultrasound and is associated with the pregnancy episode in the medical record. We excluded any deleted episode that had been entered in error as noted by a comment entered in the electronic medical record (EMR).

### Statistical Analysis

We conducted descriptive data analyses using the pregnancy episode data from our health care institution’s internal EMR system. The data included patient-specific demographic characteristics (eg, age and race), clinical characteristics (eg, parity), and socioeconomic indicators (eg, insurance status, zip code of residence). Race/ethnicity is patient-reported and documented in the EMR. Because this analysis consists of pregnancy episodes, no sex-specific analyses were performed. Baseline demographic characteristics in our obstetric population were analyzed in 2 ways: an annual comparison of pregnancy episodes between 2019 and 2020 and a comparison of new pregnancy episodes started before and after the COVID-19 mandated societal shutdown measures in March 2020. For the before and after comparisons, we defined time periods as January 1 through March 31, 2020, as the pre–societal shutdown period, and April 1 through June 30, 2020, as the post–societal shutdown period. Comparisons in demographic characteristics were compared with parametric or nonparametric tests as appropriate for continuous variables and with χ^2^ tests for categorical variables.

We analyzed the volume of initiation of new pregnancy episodes in our system compared with prior years and explored the association between the COVID-19 lockdown measures on volume of pregnancy episodes and projected future births. We identified the number of pregnancy episodes that began each week between January 2017 and March 2021 and used an interrupted time series study to characterize the differences in pregnancy starts both before and after the initiation of the spring lockdown. A Poisson regression model was used, and Fourier terms were used to adjust for annual seasonal patterns in conception, as described by Bernal et al.^8^ Because there is a delay between conception and the start of a pregnancy care episode of at least 2 weeks, we assigned the intervention binary variable of pre– and post–societal lockdown to begin March 29, 2020, 2 weeks after the start of the spring stay-at-home order, and last until June 14, 2020, 2 weeks after the end of the order. To examine whether prenatal care was being delayed during the COVID-19 pandemic, we examined the time between LMP and contact with the health care system in the time frame before the COVID-19 shutdown to the time period after the shutdown using both the Kolmogorov-Smirnov test and the Fleming-Harrington test for right-censored, 2-sampled time data. We projected future delivery volumes each month from October 2020 through February 2021 using EDDs of active pregnancy episodes with a growth rate applied, which represented the volume of patients who would deliver in an upcoming month who did not yet have a record in the EMR. This growth rate was based on data from 2019. We tested the accuracy of our birth volume projection models compared with actual birth volumes for December 2020 through February 2021.

We explored factors contributing to observed pregnancy volume changes including COVID-19 shutdown of in vitro fertilization (IVF) cycles and preterm birth rates. Rates of preterm births (ie, delivery <37 weeks’ gestation) per week were explored and quantified using changepoint analysis to determine whether an abrupt change occurred during time-series data. Preterm birth rates before and after the identified changepoint were statistically compared using the Kolmogorov-Smirnov test. Two-sided *P* < .05 were considered significant. Statistical analyses were performed with the R statistical programming language version 3.6.0 (R Project for Statistical Computing) and Microsoft Excel (Microsoft Corp). Missing data were handled in the following manner: missing race data were included in the other category, unknown Hispanic ethnicity was categorized as non-Hispanic, unknown insurance was categorized as non-Medicaid/Medicare, unknown zip codes were categorized as greater than 30 miles away, and unknown age was excluded from age analysis but included in pregnancy episode and birth projection analysis.

## Results

Table 1 shows demographic characteristics of our obstetric population from 2017 through 2020 (28 284 total pregnancy episodes; median [interquartile range {IQR}] age, 30 [27-34] years; 18 728 [66.2%] White, 3794 [13.4%] Black, and 2177 [7.7%] Asian women). Table 2 demonstrates demographic characteristics of new pregnancy episodes started in 2020 before and after the COVID-19 pandemic mandated societal shutdown. In general, our obstetric population had 15% of women older than 35 years; racial/ethnic distribution was approximately 61% White (8983 total pregnancies), 13% Black (1848 pregnancies), 7% Asian (986 pregnancies), and slightly more than 4% Hispanic (694 pregnancies) women. Approximately 40% of patients (6273 women) lived within 15 miles of the University of Michigan Hospital, and 30% (4063 women) lived more than 30 miles away from the hospital, while 24% (3469 women) had government insurance (Medicaid or Medicare). There was a statistically significant difference between the distance patients lived from our center from 2019 to 2020 (eg, >30 mi from hospital: 2094 women [28.6%] vs 1969 women [27.0%]; *P* = .002) and within 2020 before and after the COVID-19 pandemic shutdown (>30 mi from hospital: 568 [31.2%] vs 429 [27.0%]; *P* = .02).

**Table 1.** Demographic Characteristics of Obstetric Population at UMH Between 2017 and 2020

Characteristics	Pregnancy episodes, No. (%)			*P* value
	Total (n = 28 284)	2019 (n = 7311)	2020 (n = 7305)	
Maternal age, median (IQR), y	30 (27-34)	30 (27-34)	31 (27-34)	.70
Age >35 y	4310 (15.2)	1079 (14.8)	1154 (15.8)	.26
Race				
White	18 728 (66.2)	4709 (64.4)	4274 (65.7)	.09
Black	3794 (13.4)	986 (13.5)	862 (11.8)	
Asian	2177 (7.7)	527 (7.2)	459 (6.3)	
Other	3585 (12.7)	1089 (14.9)	1141 (13.4)	
Hispanic	1325 (4.7)	337 (4.6)	357 (4.9)	.45
Parity, median (IQR)	2 (1-3)	2 (1-3)	2 (1-3)	.24
Maternal zip code, distance from UMH				
0-15 mi	12 333 (43.6)	3167 (43.3)	3106 (42.5)	.002
16-30 mi	8089 (28.6)	2050 (28.0)	2230 (30.5)	
>30 mi	7862 (27.8)	2094 (28.6)	1969 (27.0)	
Insurance status				
Medicare/Medicaid	6720 (23.8)	1754 (24.0)	1715 (23.5)	.48
Non–Medicare/Medicaid	21 564 (76.2)	5557 (76.0)	5590 (76.5)	

Abbreviations: IQR, interquartile range; UMH, University of Michigan Hospital.

**Table 2.** Demographic Characteristics of Patients With New Pregnancy Episodes Started in 2020 Before and After the Onset of the COVID-19 Pandemic Societal Lockdown

Characteristics	Pregnancy episodes, No. (%)		*P* value
	Before onset of lockdown^a^ (n = 1818)	After onset of lockdown^b^ (n = 1590)	
Maternal age, median (IQR), y	30.0 (27-34)	31.0 (27-34)	.18
Age >35 y	265 (14.6)	248 (15.6)	.43
Race			
White	1194 (65.7)	1011 (63.6)	.28
Black	217 (11.9)	225 (14.2)	
Asian	129 (7.1)	114 (7.2)	
Other	278 (15.3)	240 (15.1)	
Hispanic	85 (4.7)	83 (5.2)	.51
Parity, median (IQR)	2 (1-3)	2 (1-3)	.97
Maternal zip code, distance from UMH			
0-15 mi	725 (39.9)	678 (42.6)	.02
16-30 mi	525 (28.9)	483 (30.4)	
>30 mi	568 (31.2)	429 (27.0)	
Insurance status			
Medicare/Medicaid	402 (22.1)	394 (24.8)	.07
Non-Medicare/Medicaid	1416 (77.9)	1196 (75.2)	

Abbreviations: IQR, interquartile range; UMH, University of Michigan Hospital.

^a^ Prelockdown period was January 1, 2020, to March 28, 2020.

^b^ Postlockdown period was March 29, 2020, to June 14, 2020.

**Figure 1.**
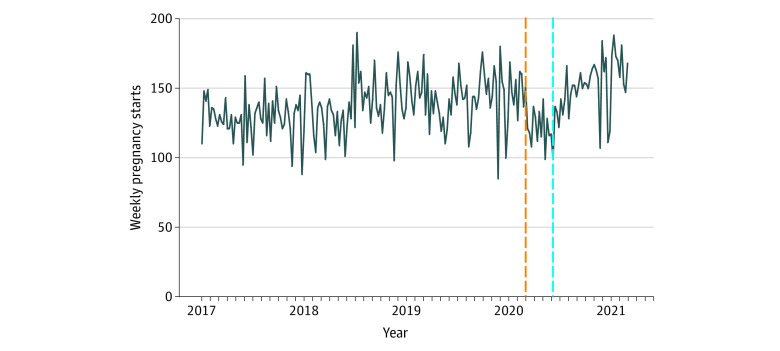
Trajectory of Weekly Volumes of New Pregnancy Episodes From 2017 to March 2021 The orange vertical dashed line indicates when the state-mandated stay-at-home order was placed, and the blue dashed line marks when the stay-at-home order was lifted.

**Figure 2.**
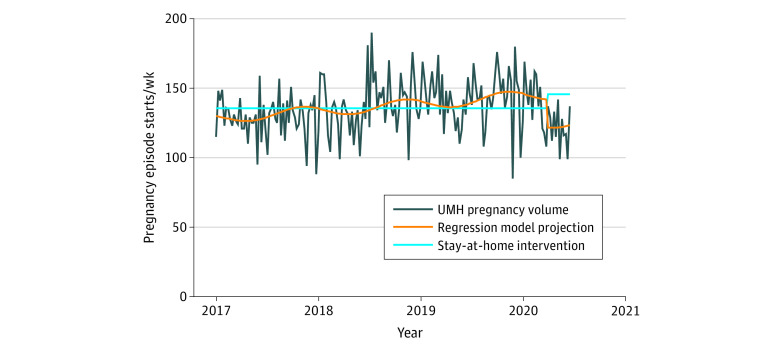
Interrupted Time Series and Poisson Regression Model of Pregnancy Episode Starts With Seasonality From 2017 to March 2021

Figure 1 shows weekly volume of pregnancy episode initiations in our health care system from 2017 through March 2021. Between the last week of March and the second week of June 2019, mean (SD) weekly pregnancy starts were 130.7 (12.4); during the same period in 2020, the weekly volume was 122.0 (14.5). Figure 2 models weekly pregnancy episode trends, accounting for seasonality, and which demonstrates an increase in the weekly number of pregnancy episode starts since 2017. Using interrupted time series to model before and after the event of the societal shutdown due to the COVID-19 pandemic, we detected a 14% reduction in pregnancy episode initiation after the societal shutdown (risk ratio, 0.86; 95% CI, 0.79-0.92; *P* < .001).

This finding of decreased pregnancy episodes could either be because of an actual decrease in initiation of prenatal care or a delay in initiation of prenatal care. To examine the possibility of delay in initiation of prenatal care, we examined the duration from LMP to first contact with our center to open the pregnancy episode. We found no change in the duration from LMP to contact with our health care center (median [IQR] duration: LMP to pregnancy start prior to pandemic lockdown, 43.5 [34-61] days vs LMP to pregnancy start after lockdown, 42.0 [33-57] days), suggesting that the observed decrease in pregnancy episodes was not associated with a delay in initiation of prenatal care.

**Figure 3.**
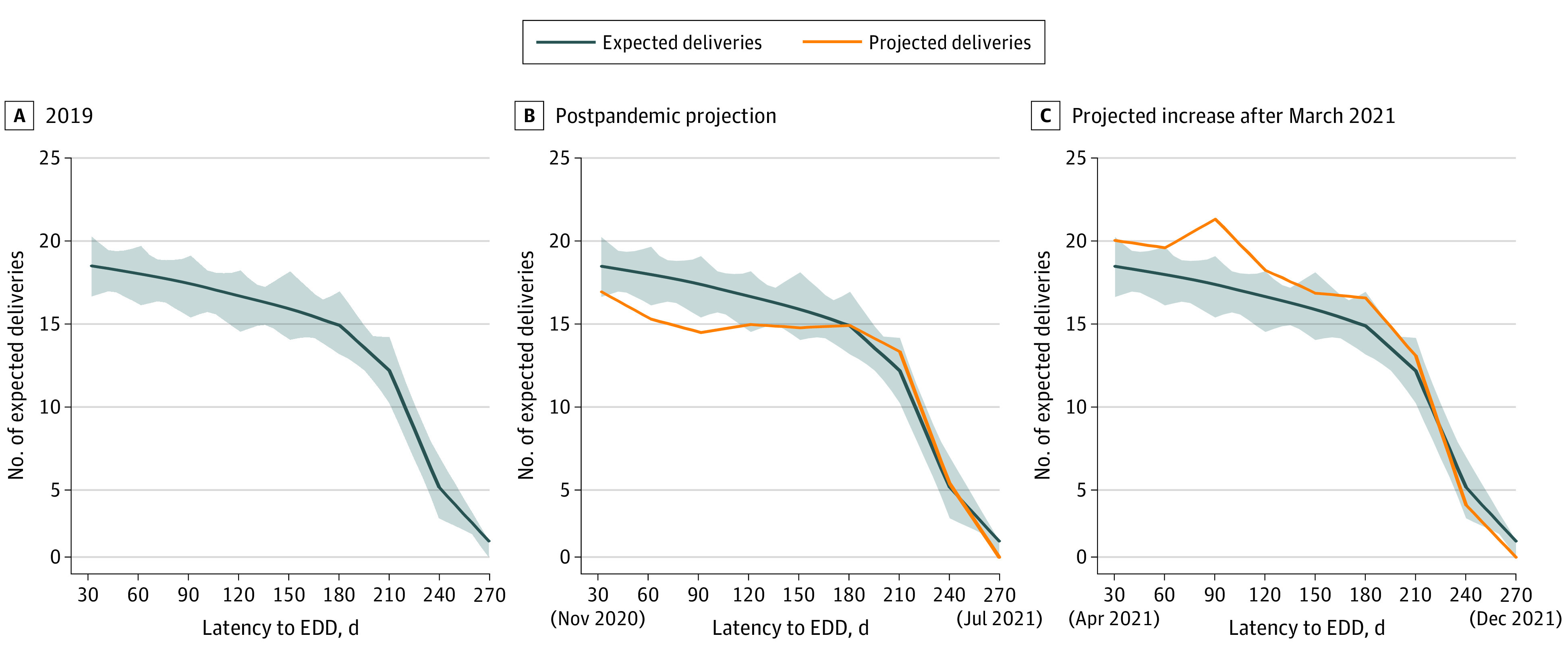
Model of Projected Deliveries Based on EDDs Known Within the University of Michigan Hospital System Gray shading represents the minimum and maximum expected deliveries, modeled with data from the University of Michigan Hospital (UMH) system from 2019. EDD indicates estimated date of delivery. Comparing the projected deliveries with the mean expected deliveries provides the expected percentage change in delivery volume relative to 2019 data.

We used our institutional EDD capacity modeling to project future delivery volumes based on pregnancy episodes in our system (Figure 3). In this modeling, we captured known EDDs within our system and calculated anticipated delivery volumes based on those EDDs. As the time latency to EDD increases, there are fewer EDDs in our system, which is expected because the early gestational ages of those pregnancies with EDDs that are more than 30 weeks away increase the proportion of EDDs that have not yet presented for care. Sixty days away from any given EDD, we are aware of 95% of patients who will deliver on a given day, and even 150 days (approximately 5 months) away from any given EDD, we have relatively complete information about projected deliveries (83% are known).

Based on our model, decreased delivery volume was expected from October 2020 through February 2021 (Figure 3). The nadir of anticipated deliveries occurred in December 2020 and January 2021, and the lower delivery volume was projected to continue until spring 2021. From the index date of October 1, 2020, we projected 16% (95% CI, −13.9% to −18.1%) fewer EDDs 60 days into the future than were seen in 2019, 18% (95% CI, −16.3% to −20.6%) fewer EDDs 90 days into the future, and 11% (95% CI, −9.6% to −14.2%) fewer EDDs 120 days into the future. When compared with actual delivery volumes relative to 2019, our projections were accurate (17.4% fewer admissions in December 2020, 16.1% fewer admissions in January 2021, and 12.2% fewer admissions in February 2021) (eTable 1 in the Supplement). The birth volume projections suggested lower than expected birth volumes to continue until April 2021. Birth volumes were projected to rebound to greater than the expected volumes based on annual trajectories, with increased birth volumes of approximately 15% (95% CI, 11%-28%) persisting until September or October of 2021 (Figure 3).

We explored factors that may have contributed to the observed and anticipated decreased delivery volumes occurring after the COVID-19 shutdown. We examined the association of closure of assisted reproduction cycles occurring March 16, as was required by state and institutional mandates, with decreased delivery volumes. We documented that there were no IVF patients who began a pregnancy episode between early April and the end of June 2020 (eFigure 1 in the Supplement). We also examined all patients who had a pregnancy episode opened within 30 days of a reproductive endocrinology visit. While the decrease in reproductive endocrinology services during the mandated shutdown contributed to overall decreased pregnancy episodes, it only explains 33% of the total decrease. We examined the association of preterm birth rates with delivery volume projections. Given that pregnancy episodes are closed after delivery occurs, if there was a sizeable increase in preterm births, pregnancy episodes would fall out of the system and could contribute to the appearance of decreased pregnancy episode volumes. We found, on the contrary, that the overall preterm birth rates decreased after the onset of the COVID-19 pandemic (eFigure 2 in the Supplement). Changepoint analysis to detect abrupt changes in time-series data determined that July 2020 represented the time point after which preterm birth rates appeared to be different. Prior to July 2020, the rate was 13.3% (or 12.0 preterm births per week of 89.8 weekly births) vs 10.2% from July to December 2020 (9.1 preterm births of 89.7 weekly births) (*P* = .001).

## Discussion

In this cohort study, we demonstrated EMR data on pregnancy episode volumes and projected birth volumes could be monitored and projected with accuracy without waiting for changes in birth volume to signal decreasing (or increasing) birth rates after major societal events. We documented an anticipated decline in births after the COVID-19 pandemic, starting in November of 2020 and persisting until spring of 2021, after which is projected a rebound in anticipated births that may exceed anticipated birth volume based on annual trajectories derived from the prior 5 years of institutional data. In our institution, we modeled pregnancy episode volume to surveil for capacity constraints and projected periods of high and low delivery volume for internal planning reasons. These same modeling techniques can be applied to estimate impacts on anticipated birth rates within a hospital or health care system, or for local/state epidemiologic surveillance. Our data suggest that the anticipated decrease in the birth rate may be best explained by lower conception rates in the weeks and months immediately following the March 2020 COVID-19 pandemic major societal shutdown. Additionally, we found that preterm birth rates may have decreased after the COVID-19 pandemic shutdown.

Pandemics and other major societal events alter population dynamics by both changing fertility rates and changing aging and death rates.^1,2,3,9,10,11^ Changing birth rates in other societal crises have been linked retrospectively to changes in economic conditions, morbidity and mortality rates among reproductive age populations, and other destabilizing societal conditions (eg, separations caused by war deployments, access to health care/contraception). Often, changes in birth rates are recorded as birth rates change, not modeled prospectively to anticipate these changes and plan accordingly.^1^ How the COVID-19 pandemic may affect birth rates has been speculated in the lay press^12,13,14,15,16,17^ but has not been fully documented, even as the societal impacts of the pandemic have persisted for longer than a year.

Population dynamics are of interest for governments, businesses, and economists because fluctuations in young and aging, workforce, and school-aged populations are critical variables in the ability to plan appropriately for social well-being, to make investments, and to anticipate economic patterns.^9,11,18,19^ In fact, encouragement of childbearing is the focus of recent government and societal policies, such as 12-month paid parental leave and other financial bonuses for childbearing in countries concerned with declining fertility rates.^18^ From a hospital perspective, planning of birth rates is important for appropriate anticipation of maternal and obstetric care needs, as well as anticipation of neonatal intensive care and pediatric subspecialty volumes.

Given the recency and evolving situation in the COVID-19 pandemic, there are few objective calculations of how it may influence birth rates globally or in the US. In June 2020, the Guttmacher Institute published survey data on how the COVID-19 pandemic is affecting female sexual and reproductive health, which found that more than 40% of women reported changing their plans about when to have children and how many children to have, with 34% wanting fewer children or to delay having children.^4^ There were notable racial and ethnic disparities, with Black and Hispanic women significantly more likely than White women to state that they were delaying childbearing or wanted fewer children. Our study documented decreased birth volume from November 2020 through February 2021 and an anticipated birth volume surge anticipated in the summer of 2021. In our institution, we do not yet see any clinically meaningful evidence of changing racial or insurance demographic distributions within the pregnant population, although our data are from a single center and do not fully capture the social consequences of the COVID-19 pandemic to its end. Similar to our findings, other centers in the US have also reported decreased preterm birth rates after the COVID-19 pandemic.^20^ A study from Smith et al^21^ estimated a potentially permanent impact on birth rates attributable just to closures of assisted reproductive technologies such as IVF, because the closures of these procedures combined with maternal aging may result in loss of fertility that will not be regained after reopening. Our study documented that decreased access to infertility treatment contributes to decreasing pregnancy rates but does not fully explain it. At this time, continued infections of and death from COVID-19, vaccine rollout initiatives, and varied societal reopening may require continued surveillance to determine the full impact of the COVID-19 pandemic on birth rates.

### Strengths and Limitations

Strengths of our study include the ability to use novel modeling techniques to project birth rate volumes with relative certainty prospectively instead of waiting until decreased birth rates are observed. Additionally, by layering on demographic characteristics, such as maternal age and race/ethnicity, assisted reproduction, and preterm birth rates, we can estimate the effects of the COVID-19 pandemic on birth demographic characteristics prospectively. We could apply changing demographic patterns within our models to anticipate fertility rates in specific patient populations, plan for high- and low-risk obstetric volumes, and plan for neonatal intensive care unit and pediatric subspecialty needs.

This study had several limitations that should be considered. First, our data are based on a single tertiary care academic center that serves both a local population as well as transported referral populations from throughout the state of Michigan. Thus, our findings may or may not be generalizable to other centers or other regions of the country. Second, we found no significant maternal demographic differences from pre– to post–COVID-19 pandemic time frames. This may be because it is too early to detect those nuanced changes or because of the specific demographic composition of our obstetric population. Third, our projections require that the pregnancy be known to the health care system. Thus, we fail to capture early pregnancy losses, terminations, or pregnancies that might be ongoing but not presenting for prenatal care.

## Conclusions

In this cohort study, we documented decreased birth rates following the COVID-19 pandemic societal changes, followed by a projected birth volume surge, suggesting that major societal changes may factor into reproductive choices. We demonstrated the use of EMR modeling to project birth rates and investigate in real-time the underlying reasons for changes in observed birth rates. These projection modeling techniques can be used in partnerships between hospitals and governmental or societal organizations to minimize the detrimental effects of the COVID-19 pandemic on society.

## Notes

Supplement. **eTable.** Projected Birth Volume Changes From EDD Modeling and Actual Birth Volume Changes **eFigure 1.** Exploration of Impact of Decreased Assisted Reproduction Procedures on Pregnancy Episode Volumes **eFigure 2.** Exploration of Preterm Birth Rates on Pregnancy Episode Volume
