# Supplementary material for: Use of Electronic Medical Records to Estimate Changes in Pregnancy and Birth Rates During the COVID-19 Pandemic
Source: JAMA Netw Open. 2021 Jun 3;4(6):e2111621. doi: 10.1001/jamanetworkopen.2021.11621 (PMC8176329; doi:10.1001/jamanetworkopen.2021.11621)
Supplement: Supplement. — eTable. Projected Birth Volume Changes From EDD Modeling and Actual Birth Volume Changes eFigure 1. Exploration of Impact of Decreased Assisted Reproduction Procedures on Pregnancy Episode Volumes eFigure 2. Exploration of Preterm Birth Rates on Pregnancy Episode Volume [file jamanetwopen-e2111621-s001.pdf]

Supplemental Online Content

Stout MJ, Van De Ven CJM, Parekh VI, et al. Use of electronic medical record to estimate changes in pregnancy and birth rates during the COVID-19 pandemic. *JAMA Netw Open.* 2021;4(6):e2111621. doi:10.1001/jamanetworkopen.2021.11621

eTable. Projected Birth Volume Changes From EDD Modeling and Actual Birth Volume Changes

eFigure 1. Exploration of Impact of Decreased Assisted Reproduction Procedures on Pregnancy Episode Volumes

eFigure 2. Exploration of Preterm Birth Rates on Pregnancy Episode Volume

This supplemental material has been provided by the authors to give readers additional information about their work.

© 2021 Stout MJ et al. *JAMA Network Open.*

eTable. Projected birth volume changes from EDD modeling and actual birth volume changes

Month of Delivery	Predicted % change versus 2019 from EDD delivery volume projections	Actual % change versus 2019
November 2020	-8.8%	-10.9%
December 2020	-15.8%	-17.4%
January 2021	-17.7%	-16.1%
February 2021	-7.4%	-12.2%

© 2021 Stout MJ et al. *JAMA Network Open.*

eFigure 1. Exploration of impact of decreased assisted reproduction procedures on pregnancy episode volumes

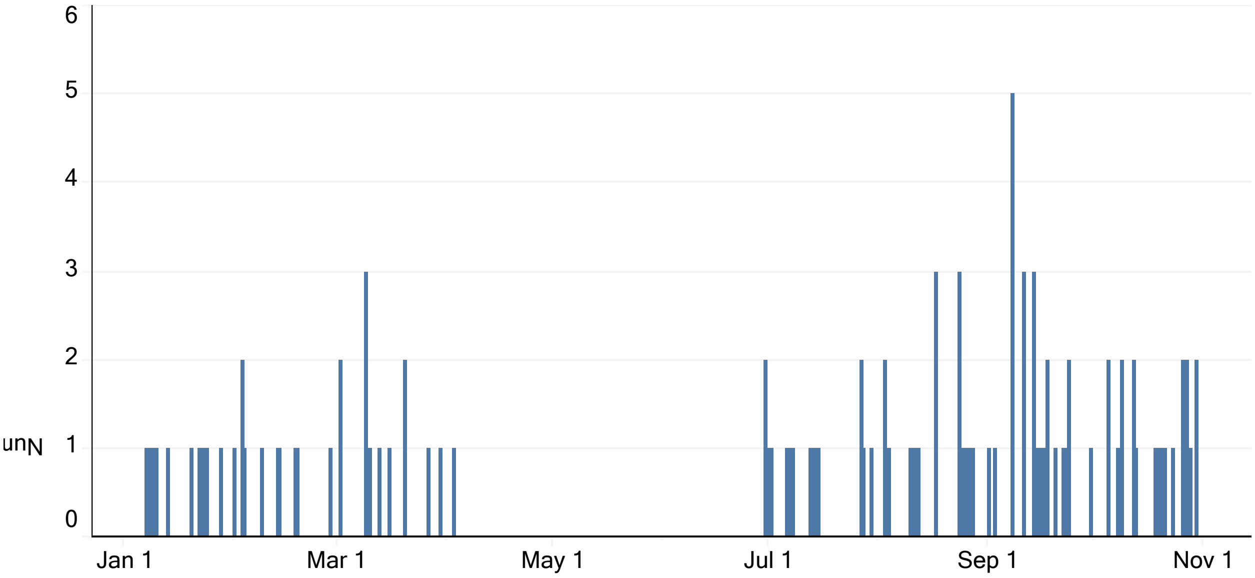

X axis is months of 2020 and Y axis is number of pregnancy episodes started per day from patients who were treated with in vitro fertilization or frozen embryo transfer (IVF/FET). There are zero pregnancy episodes resulting from IVF/FET starting in early April 2020 and continuing until the end of June 2020.

© 2021 Stout MJ et al. *JAMA Network Open.*

eFigure 2. Exploration of preterm birth rates on pregnancy episode volume

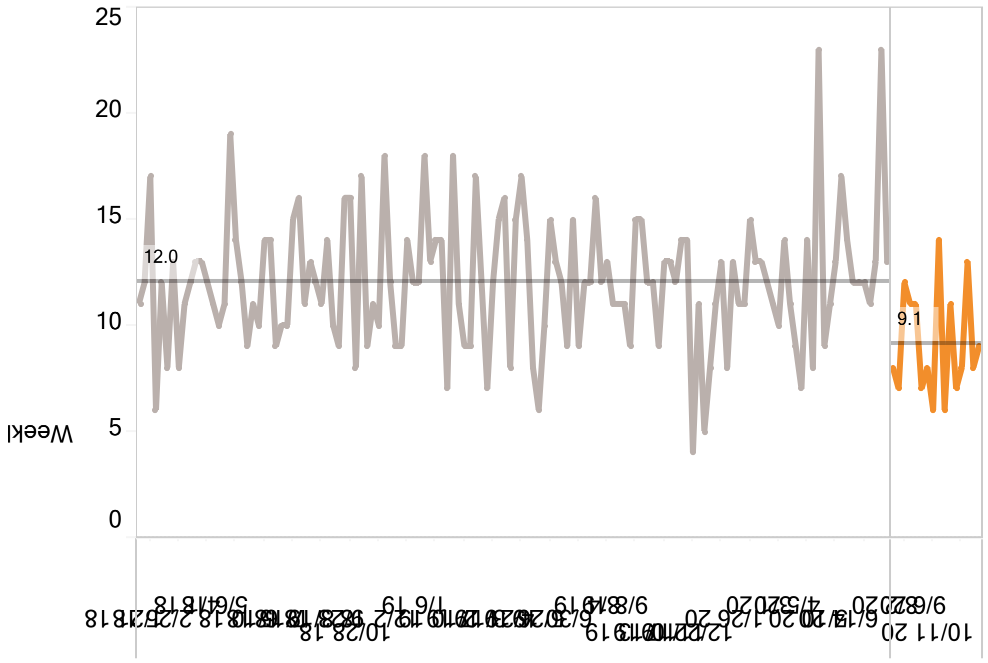

X axis is months of 2019 and 2020. Y axis is number of preterm birth per week. Changepoint analysis to detect abrupt changes in time series data identified July 2020 as the point at which the preterm birth rate pattern changes. There were an average of 12 preterm births per week before July 2020 and 9 preterm births per week after July 2020, corresponding to a PTB rate of 13.3% versus 10.2% (p<0.01).

© 2021 Stout MJ et al. *JAMA Network Open.*
